# Pharmacological Network Reveals the Active Mechanism of Qi-Replenishing, Spleen-Strengthening, Phlegm-Dispelling, and Blood-Nourishing Fufang on Coronary Heart Disease

**DOI:** 10.1155/2020/1062325

**Published:** 2020-12-29

**Authors:** Fan Zhang, Yue Liu, Sicheng Zheng, Boyi Dang, Jianan Wang, Zhe Zhang

**Affiliations:** ^1^Affiliated Hospital of Liaoning University of Traditional Chinese Medicine, Shenyang, Liaoning 110847, China; ^2^Liaoning University of Traditional Chinese Medicine, Shenyang, Liaoning 110847, China

## Abstract

This study aimed to investigate the potential targets and pathways of qi-replenishing, spleen-strengthening, phlegm-dispelling, and blood-nourishing Fufang in the treatment of coronary heart disease (CHD). The composition of Fufang was identified, followed by screening of the active components using ADME. The targets of active components were predicted and screened based on the TCMSP and BATMAN databases and were cross-validated using the CTD database and DisGeNET. A functional enrichment analysis was performed using the ClueGO + CluePedia plugins and clusterProfiler in the R package. The protein-protein interaction (PPI) network was constructed using the STRING database and Cytoscape. Finally, a pharmacological network was constructed. A total of 27 overlapping targets were obtained after cross-validation. ALB, IL-6, and TNF were the hub genes in the PPI network. The pharmacological network included 59 nodes and 189 relation pairs. Among the 59 nodes, there were 2 herbal medicine nodes (*Salvia miltiorrhiza* and *Astragalus mongholicus*), 8 chemical component nodes (magnesium lithospermate B, neocryptotanshinone II, heteratisine, daphneolone, tanshinone IIA, tanshinone IIB, soyasapogenol B, and astragaloside II), 27 target protein nodes (such as ALB, TNF, IL-6, NFKB1, APOA1, APOA2, CYP1A1, and CYP1A2), and 22 pathway nodes (such as the toll-like receptor signaling pathway, IL-17 signaling pathway, and TNF signaling pathway). Therefore, we found that the genes TNF, IL-6, NFKB1, ALB, CYP1A1, CYP1A2, APOA1, and APOA2 might be important targets of the key active compounds neocryptotanshinone II and astragaloside II. These genes targeted by the key active compounds might regulate inflammation-related pathways and the level of albumin and cholesterol in CHD.

## 1. Introduction

Cardiovascular diseases are the leading cause of deaths in the world, accounting for more than twice the number of deaths caused by cancers, and have been a huge economic burden on the healthcare and society [1]. Coronary heart disease (CHD) is the most common cardiovascular disease, characterized by the formation of atherosclerotic plaques [2]. Risk factors for CHD include various aspects, aside from age and gender, such as dyslipidemia, hypertension, nutritional disorders, obesity, diabetes, and smoking [3]. The major cause of CHD is coronary atherosclerosis, which narrows or progressively occludes the lumen of the blood vessels, resulting in acute and temporary ischemia and hypoxia of the myocardium [4]. It was named “thoracic obstruction” according to the traditional Chinese medicine (TCM) theory [5].

TCM has been used in the treatment of CHD for thousands of years and has achieved certain curative effects [6, 7]. Studies have reported the roles of TCM. For example, based on a blinded randomized controlled trial, Chen et al. showed that Huoxue Huayu therapy is an effective and safe therapy for patients with CHD after percutaneous coronary intervention; it works by decreasing the degree of restenosis and improving the revascularization rate [8]. Liu et al. suggested that Radix *Salvia miltiorrhiza* compounds could decrease the risk of CHD by improving the biochemical indices (reducing the levels of triglycerides, cholesterol, and lipids, while increasing the levels of apolipoprotein A/B/E and total bilirubin) of patients with CHD [9]. In addition, Gong et al. found that Radix *Salvia miltiorrhiza*, Rhizoma Chuanxiong, Radix Astragali, and other herbs were frequently used for the treatment of CHD as evidenced by a literature review covering the recent 20 years [5]. Although many TCMs have been reported to show curative effects when used for CHD, their widespread applications are also hampered due to a lack of rigorous clinical trials and their complex compositions, as well as their unclear pharmacological mechanisms.

Based on the TCM theory, the spleen is connected to the heart meridians, and their functions are compatible, so the malfunctioning of the spleen can lead to changes in the heart function. Guanlin Yang proposed the method of “Treatment According with the Spleen” and developed a qi-replenishing, spleen-strengthening, phlegm-dispelling, and blood-nourishing (QSBD) TCM granule for the treatment of CHD [10–12]. They found that the QSBD TCM granule showed obvious effects on stable angina pectoris associated with CHD. However, its pharmacological mechanisms are still unclear. Network pharmacology refers to the integrated analysis of a system biology network and pharmacology, which is in accordance with the core concept of the holistic philosophy of TCM, and provides insight into the detailed compound-target and target-pathway relationships of herbs used to treat complicated diseases [13–15]. Therefore, the pharmacological network of the QSBD TCM granule on CHD was investigated in this study to further uncover its potential targets and pathways.

## 2. Materials and Methods

### 2.1. Candidate Components in QSBD TCM Granules

Information from all components of the four main Chinese medicinal herbs in the QSBD TCM granule (including *Astragalus mongholicus*, bighead atractylodes rhizome, Rhizoma Pinelliae, and *Salvia miltiorrhiza*) was retrieved from the TCMSP database [16] (http://lsp.nwu.edu.cn/browse.php?qc=herbs), including molecule name, molecular weight, AlogP, Hdon/Hacc, oral bioavailability (OB), Caco-2, blood-brain barrier, drug likeness (DL), and drug half-life. Supplemental Figure 1 shows the workflow of this study.

### 2.2. Screening Effective Composition of Herbs

All the components were screened using the absorption, distribution, metabolism, and excretion (ADME) model [17] from the TCMSP database. OB is one of the most important pharmacokinetic parameters, and components with more than 30% OB are considered to have high bioavailability, a crucial index in evaluating the DL of a potentially effective component. Compounds with a threshold of OB ≥ 30 and DL ≥ 0.18 were screened as effective components.

### 2.3. Prediction of Drug Targets

The protein targets of the effective components were predicted using the TCMSP database and the BATMAN-TCM database [18] (http://bionet.ncpsb.org/batman-tcm/), and the targets with similarity scores >10 were screened.

### 2.4. Cross-Validation of Target Proteins

To validate the target proteins and to narrow the range of possible target proteins, “coronary heart disease” was used as the keyword to search CHD-associated genes from the Comparative Toxicogenomics Database (CTD) [19] (http://ctdbase.org/, updated 2018), and the genes with inference scores >40 were selected. In addition, “coronary heart disease” was also used as the keyword to search CHD-associated genes from the DisGeNET database (version 6.0) [20]. The overlaps between the CHD-associated genes and the predicted protein targets were selected and used in the subsequent analyses.

### 2.5. Functional Enrichment Analysis

The gene ontology (GO) functional network analysis was conducted using the ClueGO + CluePedia plugins [21] of Cytoscape with the parameter setting as biological process, adjusted *P* value ≤0.01, and default GO level (global option GO levels 3–8) [22]. The GO functional network was visualized using Cytoscape. The Kyoto Encyclopedia of Genes and Genomes (KEGG) pathways were enriched using clusterProfiler in the R package, and the significant pathways were selected with *P* < 0.01.

### 2.6. Construction of the Protein-Protein Interaction Network

The protein-protein interactions (PPIs) were retrieved from the STRING database (version: 10.0, http://www.string-db.org/) with a required confidence (combined score) >0.9. The PPI network was visualized using Cytoscape based on the retrieved interactions.

### 2.7. Construction of the Pharmacological Network

In order to further characterize the molecular mechanisms of the effective components of the QSBD TCM granule on CHD, a pharmacological network was constructed using Cytoscape based on the herbal medicine-component-target protein-pathway data.

### 2.8. Molecular Docking

The three-dimensional (3D) structures of the key target proteins were downloaded from the RCSB Protein Data Bank (http://www.rcsb.org/) and were modified by removing the ligand and water molecules, adding nonpolar hydrogens, amino acid optimization, and calculating Gasteiger charges using AutoDock 4.2 software. The SDF structures of the compounds were downloaded from the PubChem Compound database (https://pubchem.ncbi.nlm.nih.gov/), and the data were converted into a PDB format using PyMOL (version 2.0, Schrödinger, LLC.) for molecular docking simulations. Molecular docking simulations were performed using AutoDock 4.2.6 with the compounds as ligands.

## 3. Results

### 3.1. Identification of the Effective Components in QSBD TCM Granules

The number of chemical constituents in the four main Chinese medicinal herbs in the QSBD TCM granule was identified. Among them, 87 chemical components were identified from *Astragalus mongholicus*, where 20 effective components were screened with an OB ≥ 30 and DL ≥ 0.18. *Salvia miltiorrhiza* had more chemical components (202) compared to the other herbs (bighead atractylodes rhizome, 55; Rhizoma Pinelliae, 116). There were 65 effective components for *Salvia miltiorrhiza*, 7 effective components for bighead atractylodes rhizome, and 13 effective components for Rhizoma Pinelliae that had an OB ≥ 30 and DL ≥ 0.18.

### 3.2. Prediction and Screening of Drug Targets

Based on the BATMAN online tool, 222 important components with target records were predicted for the four main Chinese medicinal herbs in the QSBD TCM granule (Supplemental Table 1), of which 31, 81, 75, and 35 important components with target records were predicted for Rhizoma Pinelliae, bighead atractylodes rhizome, *Salvia miltiorrhiza*, and *Astragalus mongholicus*, respectively. After screening important components using ADME and targets with scores >10, a total of 6 important components (magnesium lithospermate B, neocryptotanshinone II, heteratisine, daphneolone, tanshinone IIA, and tanshinone IIB) with 275 targets were obtained for *Salvia miltiorrhiza*, and 2 important components (soyasapogenol B and astragaloside II) with 15 targets were obtained for *Astragalus mongholicus* (Supplemental Table 2).

### 3.3. Cross-Validation of Targets

**Figure 1:**
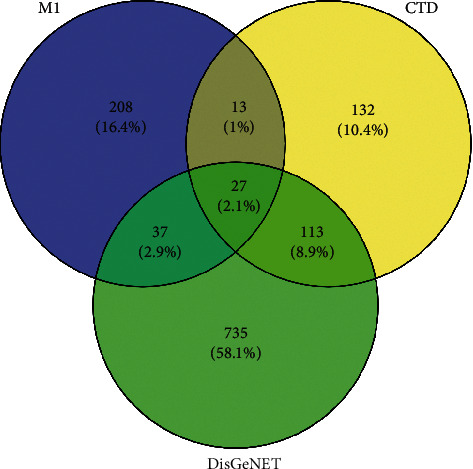
Venn diagrams for cross-validation targets.

A total of 285 CHD-associated genes with an inference score >40 were predicted from the CTD database, and 912 CHD-associated genes were predicted from the DisGeNET database. In all, 27 overlapped genes were obtained between the CHD-associated genes and the predicted targets (Figure 1). The 27 genes were used in the subsequent analyses.

### 3.4. Functional Enrichment Analysis

**Figure 2:**
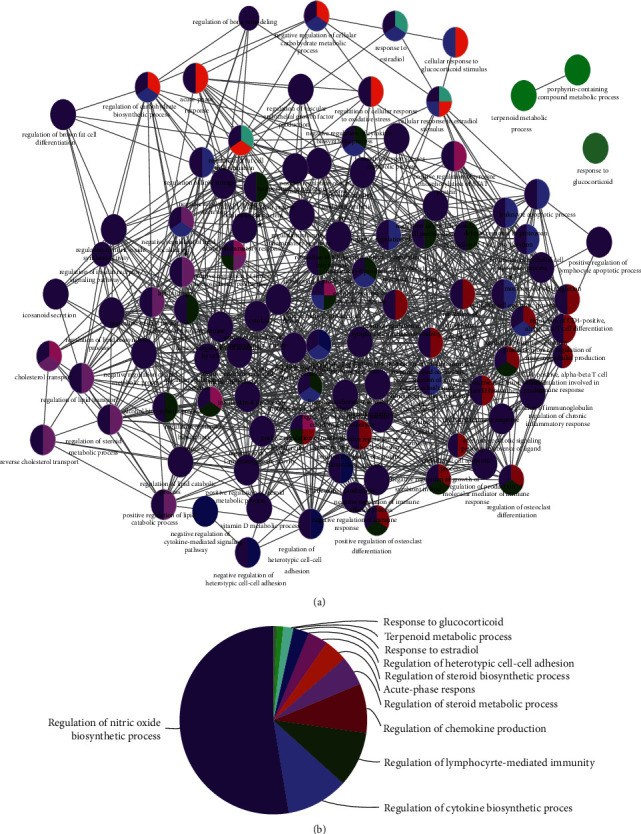
The results of gene ontology (GO) functional analyses. (a) GO functional network for the 27 target genes. The nodes represent GO functional terms; larger nodes represent more significant *P* values; lines represent the correlation between functional terms. (b) Pie chart showing the clusters of the GO functional terms.

**Figure 3:**
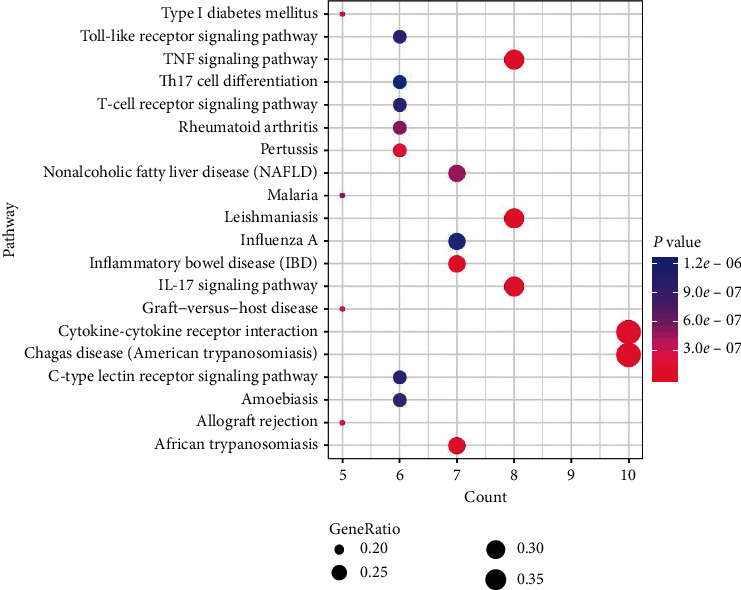
Bubble chart of top 20 pathways. Larger dots represent a larger proportion of the number of enriched genes to the total number of genes; the color gradient from blue to pink represents decreasing *P* values.

The results of GO functional analysis are shown in Figure 2. The 27 genes were significantly enriched in 98 GO functional terms (Figure 2(a) and Supplemental Table 3), including response to glucocorticoids, porphyrin-containing compound metabolic process, and terpenoid metabolic process. The 98 functional terms were classified into 11 categories based on the kappa coefficient and include regulation of the nitric oxide biosynthetic process, regulation of the cytokine biosynthetic process, regulation of lymphocyte-mediated immunity, and other biological processes (Figure 2(b)). In addition, the 27 genes were significantly enriched in 63 KEGG pathways (Supplemental Table 4). The top 20 pathways are displayed in Figure 3, showing cytokine-cytokine receptor interactions, the TNF signaling pathway, and the IL-17 signaling pathway, among others.

### 3.5. PPI Network Analysis

**Figure 4:**
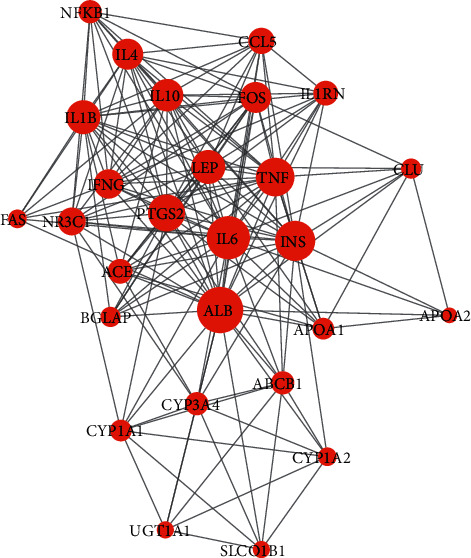
The protein-protein interaction (PPI) network. The nodes represent target proteins; the node size represents the degree of each node; lines represent the interactions between two nodes.

Proteins and their functional interactions are at the core of cellular processes, and their connectivity network helps to understand the cellular function and biological phenomena from a system-wide level [23]. The STRING database allows the construction of a comprehensive and objective PPI network involving direct (physical) and indirect (functional) interactions. The PPI network consisted of 27 nodes and 172 interactions (Figure 4), of which albumin (ALB, degree = 25), interleukin 6 (IL-6, degree = 23), insulin (INS, degree = 21), tumor necrosis factor (TNF, degree = 20), and prostaglandin-endoperoxide synthase 2 (PTGS2, degree = 19) were the main hub proteins.

### 3.6. Construction of a Pharmacological Network

**Figure 5:**
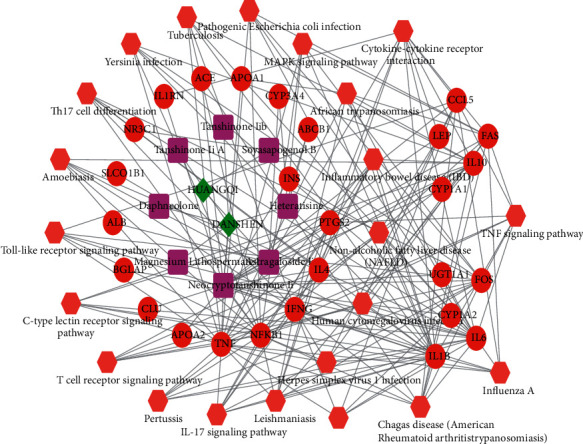
The pharmacological network. The diamonds represent herbal medicines; squares represent chemical components; circular nodes represent target genes; hexagons represent pathways. HUANGQI, *Astragalus mongholicus*; DANSHEN, *Salvia miltiorrhiza*.

The pharmacological network included 59 nodes and 189 relation pairs (Figure 5). Among the 59 nodes were 2 herbal medicine nodes (*Salvia miltiorrhiza* and *Astragalus mongholicus*), 8 chemical component nodes (magnesium lithospermate B, neocryptotanshinone II, heteratisine, daphneolone, tanshinone IIA, tanshinone IIB, soyasapogenol B, and astragaloside II), 27 target protein nodes (such as TNF, IL-6, NFKB1, APOA1, APOA2, CYP1A1, and CYP1A2), and 22 pathway nodes (such as toll-like receptor signaling pathway, IL-17 signaling pathway, and TNF signaling pathway).

### 3.7. Results for Molecular Docking

**Figure 6:**
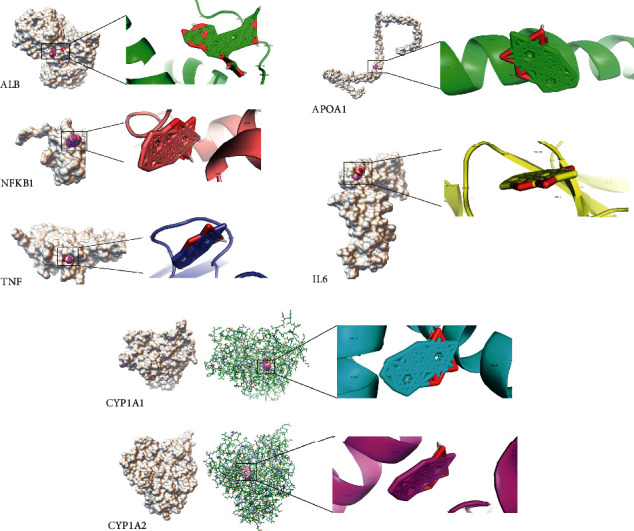
Molecular models of astragaloside II and neocryptotanshinone II binding to their predicted protein targets. ALB protein showed interaction with the astragaloside II molecule. The proteins CYP1A1, CYP1A2, APOA1, TNF, IL-6, and NFKB1 showed interactions with the neocryptotanshinone II molecule. Off-white diagrams represent receptors (target proteins), and pink diagrams represent ligands (compounds).

Molecular docking was performed to verify the key compound-target interactions in the above analysis, including the key compounds neocryptotanshinone II and astragaloside II and key targets CYP1A1, CYP1A2, APOA1, TNF, IL-6, NFKB1, and ALB. The 3D structures of these key compounds and target proteins are shown in Supplemental Figure 2. For the docking results, a total of 10 docking models were obtained, and the docking model with the best affinity (lowest binding energy) was selected as the optimal docking model. The results showed that astragaloside II had a strong affinity with ALB protein. Neocryptotanshinone II had strong affinity with multiple proteins, of which it showed the best affinity with TNF (Table 1, Figure 6).

## 4. Discussion

The TCM is a complex system composed of many compounds, showing complicated pharmacological activities (synergistic or antagonistic) with multiple targets and pathways. Network pharmacology meets the core concept of the holistic philosophy of TCM and has been widely used to investigate the detailed compound-target and target-pathway relationships of herbs used for the treatment of complicated diseases [24–26]. In the current study, two key herbal medicines (*Salvia miltiorrhiza* and *Astragalus mongholicus*), corresponding to 8 key chemical components, were identified, and these chemical components were found to target 27 genes and major gene-regulated pathways.

The recruitment of inflammatory cells to injured vascular tissues could contribute to the formation of plaques by secreting inflammatory mediators. Therefore, inflammatory markers have been considered as risk factors for CHD [4, 27]. IL-6 is an inflammatory cytokine secreted by activated macrophages and lymphocytes; the risk factors of CHD might elevate IL-6 levels. Social disruption stress showed effects on the increase of atherosclerotic plaque areas in apolipoprotein E^−/−^ mice, probably by upregulating IL-6 levels [28]. Danesh et al. showed that there were significant correlations between elevated IL-6 levels and increased CHD risk, suggesting an underlying relevance of pathways involving IL-6 in the pathogenesis of CHD [29]. Hou et al. indicated that circulating IL-6 was associated with different kinds of cardiovascular diseases and that IL-6 showed an effect on platelets which were essential for blood coagulation [30]. Hot et al. demonstrated that IL-17 coupled with TNF*α* had a synergistic induction on the generation of IL-6 and had significant procoagulant and prothrombotic effects in blood vessels [31]. In this study, several genes (TNF, IL-6, NFKB1, etc.) regulated by neocryptotanshinone II were implicated in inflammation-related pathways, such as the IL-17 signaling pathway, TNF signaling pathway, and Th17 cell differentiation pathway. Therefore, we speculated that neocryptotanshinone II exerted pharmacological activities on CHD by regulating inflammatory cytokines and their signaling pathways.

Albumin (ALB) is a 69 kDa protein that normally accounts for more than 50% of the total plasma protein content, functioning in regulating blood plasma colloid osmotic pressure, and acts as a carrier protein [32]. Low levels of serum ALB have been found to be associated with different kinds of cardiovascular diseases, including venous thromboembolism, CHD, and incident ischemic heart disease [33]. Chien et al. indicated that low levels of serum ALB were related to an increase in hard cardiovascular events and all-cause mortality for patients with stable CHD, suggesting the worse prognosis of low levels of serum ALB in stable CHD [34]. It was reported that decreased serum ALB levels may indicate worse prognosis of patients with CHD, probably by increasing autophagy [35, 36]. In our study, ALB was a hub gene with the highest degree in the PPI network and was a target gene of astragaloside II in the pharmacological network. Astragaloside II is an active compound from the medicinal herb Radix Astragali and has been reported to enhance T-cell activation [37], inhibit autophagy of hepatocellular carcinoma cells [38], and enhance the secretion of adiponectin in primary adipocytes [39]. Here, we suggested that astragaloside II played roles in the treatment of CHD by targeting ALB and regulating the level of ALB in blood.

An elevated low-density lipoprotein cholesterol level is one of the major causes of CHD and is an indispensable and independent index to predict the risk of CHD [40]. Cytochrome P450 family 1 subfamily A member 1 (CYP1A1) and CYP1A2 encode members of the cytochrome P450 superfamily of enzymes, which catalyze multiple reactions associated with drug metabolism, as well as the synthesis of cholesterol and steroids [41–43]. Apolipoprotein A-I (APOA1) and apolipoprotein A-II (APOA2) are major protein components of high-density lipoprotein (HDL) particles in plasma, functioning in the transportation of cholesterol on HDL [44]. Human and recombinant APOA1 have been reported to promote HDL-mediated cholesterol efflux and improve atherosclerosis [45, 46]. In this study, CYP1A1, CYP1A2, APOA1, and APOA2 were all targets of neocryptotanshinone II in the pharmacological network. Neocryptotanshinone II is the active compound from the medicinal herb *Salvia miltiorrhiza*. Liu et al. suggested that Radix *Salvia miltiorrhiza* compounds could decrease the risk of CHD by improving the biochemical indices (reducing the levels of triglycerides, cholesterol, and lipids, while increasing the levels of apolipoprotein A/B/E and total bilirubin) of patients with CHD [9]. Therefore, we concluded that neocryptotanshinone II played roles in the treatment of CHD by targeting CYP1A1, CYP1A2, APOA1, and APOA2 to regulate cholesterol levels.

## 5. Conclusions

Network pharmacological analysis revealed that 8 key compounds in *Salvia miltiorrhiza* and *Astragalus mongholicus* targeted 27 genes and the biochemical pathways in CHD they are involved in. The genes TNF, IL-6, NFKB1, ALB, CYP1A1, CYP1A2, APOA1, and APOA2 might be important targets of the key active compounds neocryptotanshinone II and astragaloside II. The genes targeted by these key active compounds might regulate inflammation-related pathways and the level of albumin and cholesterol in CHD.

## Figures and Tables

**Table 1:** The binding energies of the optimal docking models.

Compounds	Target proteins	Binding energy	Cluster RMSD	Reference RMSD
Astragaloside II	ALB	−12.27	0.00	12.30
Neocryptotanshinone II	APOA1	−6.80	0.00	111.46
Neocryptotanshinone II	CYP1A1	−9.50	0.00	45.40
Neocryptotanshinone II	CYP1A2	−9.28	0.00	35.30
Neocryptotanshinone II	IL-6	−7.45	0.00	92.33
Neocryptotanshinone II	NFKB1	−7.15	0.00	14.42
Neocryptotanshinone II	TNF	−9.53	0.00	23.12

## Data Availability

All the data generated or analyzed during this study are included in this published article.

## Supplementary Materials

Supplementary Materials Supplemental Table 1: the 222 components with target records for the four main Chinese medicinal herbs. Supplemental Table 2: the component-target pairs for the important components as screened by ADME. Supplemental Table 3: enriched GO functional terms for the 27 target genes. Supplemental Table 4: enriched KEGG pathways for the 27 target genes.
