# Supplementary material for: Pharmacological Network Reveals the Active Mechanism of Qi-Replenishing, Spleen-Strengthening, Phlegm-Dispelling, and Blood-Nourishing Fufang on Coronary Heart Disease
Source: Evid Based Complement Alternat Med. 2020 Dec 29;2020:1062325. doi: 10.1155/2020/1062325 (PMC7785359; doi:10.1155/2020/1062325)
Supplement: Supplementary Materials — Supplemental Table 1: the 222 components with target records for the four main Chinese medicinal herbs. Supplemental Table 2: the component-target pairs for the important components as screened by ADME. Supplemental Table 3: enriched GO functional terms for the 27 target genes. Supplemental Table 4: enriched KEGG pathways for the 27 target genes. [file 1062325.f1.zip › Supplementary files/Supplementary figures.doc]

**Supplemental Figure 1.** The workflow of this study.

## Supplemental Figure 2. The 3D structures of the key compounds and target proteins. In the 2D structures of compounds, green represents carbon, gray represents hydrogen, and red represents oxygen.
